# Insights into lipid metabolism and immune-inflammatory responses in the pathogenesis of coronary artery ectasia

**DOI:** 10.3389/fphys.2023.1096991

**Published:** 2023-01-25

**Authors:** Li Jiang, Wei Wei, Sheng Kang, Xiao-Lin Li, Yu Luo

**Affiliations:** ^1^ Department of Cardiovascular Medicine, East Hospital, Tongji University School of Medicine, Shanghai, China; ^2^ Department of Cardiovascular Medicine, Jian East Hospital, Jinggangshan University School of Medicine, Jiangxi, China

**Keywords:** coronary artery ectasia, lipid metabolism, cytokine, immune-inflammatory response, pathogenesis

## Abstract

Coronary artery ectasia (CAE) is a rare finding that is associated with poor clinical outcomes (Kawsara et al. 2018), and disorders in lipid metabolism have been reported in CAE. Lipids constitute one of the three metabolite types that regulate bodily functions and are also powerful signaling molecules (Han 2016; Zhu et al. 2021) that affect immunoregulation and inflammatory responses *via* a series of transcription factors and signaling pathways (Barrera et al. 2013). Although abnormal lipid metabolism and immunoinflammatory responses have been reported in CAE, their roles in the pathogenic mechanisms underlying CAE are currently unclear.

## Introduction

**FIGURE 1:**
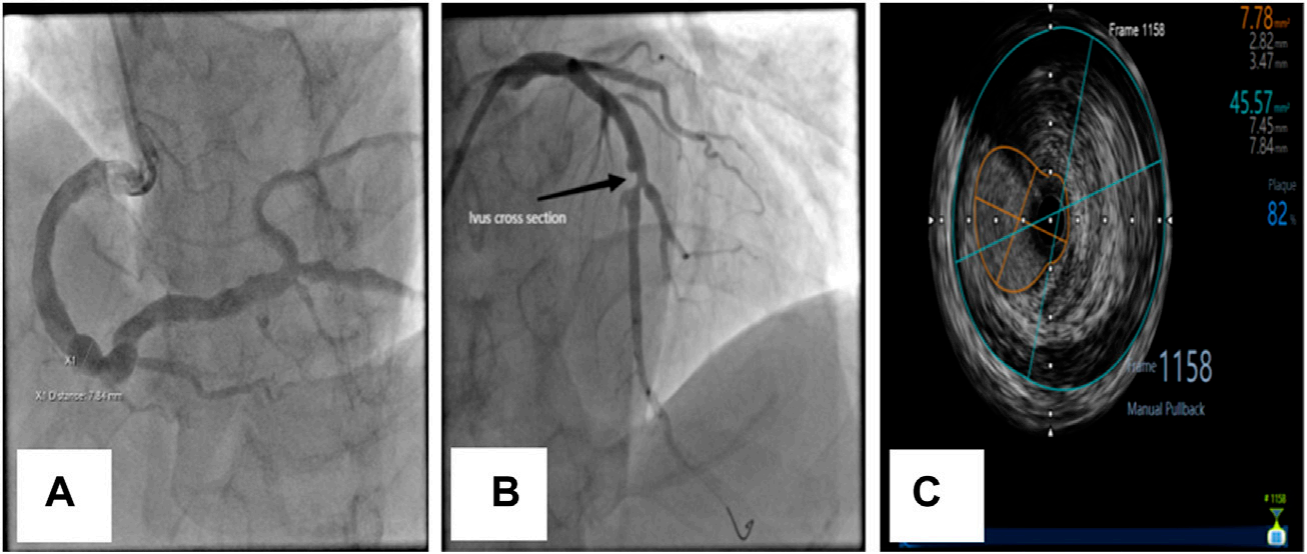
Coronary angiographic and intravascular ultrasonographic (ivus) images of CAE. **(A)** Diffuse dilation of the right coronary artery. **(B)** Diffuse dilation with significant atherosclerosis and stenosis of the left anterior descending (LAD) coronary artery in the same patient. **(C)** Ivus image of LAD.

**FIGURE 2:**
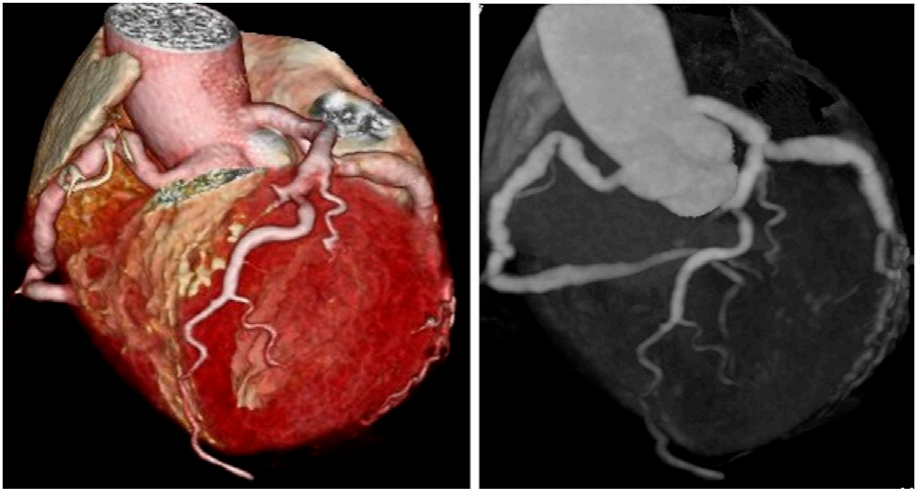
Two types of coronary CT angiography (volume-reproduction model) show diffuse coronary ectasia.

Coronary artery ectasia (CAE) is reported in 1.5–5% of patients who have undergone coronary angiography (Brunetti et al., 2014). CAE is defined as dilatation of an arterial segment diameter at least 1.5 times that of an adjacent natural artery and involves at least one-third of the relevant artery (Ozturk et al., 2015) (Figure 1). The terms CAE and coronary artery aneurysm (CAA) are frequently used interchangeably, however, they carry distinct phenotypes and definitions. CAE is characterized by dilatation of an arterial segment with at least one-third of the involved artery, whose diameter is at least 1.5 times of a proximal natural artery. Conversely, CAA is a focal-appearing dilatation. In this article, CAE refers to the diffused coronary dilatation with or without stenosis. Although CAE is a specialized form of traditional atherosclerotic coronary artery disease (CAD), it differs from CAD in many ways, as depicted in Table 1. In addition, coronary CT angiography is today an alternative diagnostic strategy (Figure 2). In contrast to its stenotic counterpart (i.e., CAD), CAE has been studied relatively infrequently. CAE encompasses clinical presentations and implications similar to those of CAD, including stable angina and acute coronary syndrome (ACS) that result from either coronary thrombus formation or impaired coronary blood flow due to dilated coronary arteries (Mavrogeni 2010). The key mechanism underlying CAE, however, has not been fully identified. In light of previous reports, it has been suggested that there may be more than one mechanism involved (Table 2). We herein evaluated lipid metabolism and immune-inflammatory responses that have been studied extensively in the pathogenesis of CAE.

**TABLE 1:** Major Similarities and differences between CAE and coronary artery disease.

	Coronary artery disease	Pure-CAE
Epidemiology	about 10% of the population	1.5%–5% undergo coronary angiography
Gender orientation	men more than women	men more than women
EEM area	normal	expanded
Effective lumen	narrow	expanded
Most affected artery	no difference among the three arteries	right coronary
Classification	single branch or multiple branch lesions	Markis I,II,III,IV
Chest tightness and chest pain	common	uncommon
Myocardial infarction	may be a cause	may be a cause
Slow coronary flow	visible when the coronary artery is nearly occluded	universal phenomenon
Kawasaki disease	irrelevant	related
Peripheral aneurysms	irrelevant	related
Collagen vascular diseases	irrelevant	related
Varicocele	irrelevant	related
Herbicide spray exposure	irrelevant	related
Infections	not sure	related
Coronary fistula	irrelevant	related
Cocaine abuse	irrelevant	related
Coronary anomalies	irrelevant	related
Hereditary collagenosis	irrelevant	related
Connective tissue diseases	irrelevant	related
Autoimmune disease	irrelevant	related
Carotid intima-media thickness	related	irrelevant
Family history	related	not sure
Hypertension	related	related
Diabetes mellitus	related	negative correlation
Hyperlipidemia	related	related
Treatment		
PCI	effective	invalid
CABG	effective	invalid
Surgical resection	invalid	effective for aneurysmal ectasia
Covered stent	invalid unless the main branch is perforated	effective for aneurysmal ectasia
Coil embolization	invalid unless the side branch is perforated	effective for aneurysmal ectasia
Anticoagulation	not applied unless concomitant with atrial fibrillation and/or other disease	effective
Antiplatelet	effective	effective
Statin	effective	effective

CAE, coronary artery ectasia; EEM, external elastic membrane; PCI, percutaneous coronary intervention; CABG, coronary artery bypass grafting.

**TABLE 2:** Potential Causes and Pathogenic Mechanisms underlying CAE.

Cause	Pathogenic mechanism of coronary ectasia
Atherosclerosis	Local mechanical stress from stenosis, enhanced inflammatory response-induced proteolysis of extracellular matrix proteins
Kawasaki disease	Autoimmunity, vasculitis
Genetic susceptibility	Specific HLA class II genotypes such as HLA-DR B1*13 are more detectable
Inflammatory disorders (vasculitis)/connective tissue disorders	Increased plasma levels of intercellular adhesion molecule-1, vascular cell adhesion molecule-1, and E-selectin; imbalances in protein levels of matrix metalloproteinase and its tissue inhibitor
Coronary Fistula	Compensatory dilatation secondary to a high-flow state
Coronary anomalies	Compensatory dilatation secondary to (e.g., ALCAPA) myocardial ischemia
Infection	Direct pathogen invasion of arterial wall, immune complex deposition
Trauma/iatrogenic	Mechanical and shear wall stress, and non-healing dissections
Drug-related	Vasoconstriction/endothelial damage

HLA, human leukocyte antigen; ALCAPA, anomalous origin of the left coronary artery from the pulmonary artery.

## Role of lipids in the pathogenesis of CAE

In a report of familial hypercholesterolemia (FH), investigators described for the first time the association between plasma lipoproteins and coronary artery aneurysm. A 23-year-old male patient with homozygous familial hypercholesterolemia was confirmed with coronary artery dilatation by coronary angiography, and the patient presented with systemic xanthomatosis and severe hyper-low-density lipoproteinemia (Mabuchi et al., 1986; Genda et al., 1987). Another study revealed that repeated plasma exchange reduced serum low-density lipoprotein cholesterol (LDL-C) levels in heterozygous FH, resulting in angiographic improvements in CAE (Thompson et al., 1980). In a study of 197 asymptomatic hypercholesterolemic (FH) subjects, Sudhir et al. examined the prevalence of CAE and its association with coronary artery risk factors, and their results showed that the incidence of CAE was significantly higher in the FH population than in the control group and that it was associated with lower high-density lipoprotein cholesterol (HDL-C) levels (*p* = 0.003) and higher LDL-C/HDL-C ratios (*p* = 0.003) (Sudhir et al., 1995). Based on these studies, higher LDL-C levels, lower HDL-C levels, and a higher LDL/HDL ratio are now considered to reflect value in the prediction of the onset and development of CAE.

Collagen and elastin fibers in the dilated coronary segment were previously shown to be significantly degraded, with the inner and outer elastic layers destroyed, but there was no evidence of dilation found in the intact and uninjured sites of the media. This suggested that enzymatic degradation of the media may constitute a key point in the pathogenesis of CAE (Daoud et al., 1963; Markis et al., 1976; Swanton et al., 1978). At the molecular level, LDL-C is bound to elastin, collagen, and proteoglycan through oxidative modification that enhances its affinity for matrix components (Galis et al., 1995). The oxidized LDL-C is subsequently engulfed by macrophages and smooth muscle cells to develop the foam cells that enhance the active breakdown of the extracellular matrix by their production of matrix-degrading enzymes—including MMP-2, MMP-9, and MMP-12. These actions then ultimately lead to coronary artery dilation (Gertz et al., 2015) (Antoniadis et al., 2008).

Based on conventional lipid components, recent studies revealed that two phospholipid species in CAE, sphingomyelin (SM) and phosphatidylcholine (PC), were significantly downregulated compared with healthy controls (Boles et al., 2017). PC exerts the important action of carrying fatty acids and portrays an intermediate role in lipid metabolism; thus, PC expression profiles provide critical information on lipid regulation and disease effects (Wymann and Schneiter 2008). SM participates in the formation of coronary artery dilation by inducing atherosclerotic lipoproteins to infiltrate the arterial wall, stimulating lipoprotein aggregation and macrophage foam-cell formation (Stegemann et al., 2011).

CAE can be observed in herbide spray exposure, hereditary collagenosis, connective tissue diseases, autoimmune disease, etc. This demonstrates that inflammation is primary etiology of CAE, which is close linked to inflammatory response. From the pathogenesis of Kawasaki disease, inflammatory response induced CAE without the involvement of lipid metabolism disorder, which is a prerequisite for the development of CAE. Aberrant lipid metabolism will aggravate inflammatory response. Instead of CAE, Simple lipid metabolic disorders frequently result in coronary atherosclerosis. It is speculated that aberrant lipid metabolism may trigger CAE through inflammatory response or other mechanisms, which is neither a required or sufficient condition for CAE (Zhu et al., 2021).

## Role of inflammation and related markers in the pathogenesis of CAE

The argument that CAE originates from a chronic inflammatory state has attracted wide attention in recent years, and chronic inflammatory mediators such as cytokines, proteolytic enzymes, growth factors, cell adhesion molecules, and systemic inflammatory mediators are known to be involved in its pathogenesis.

### Role of adhesion molecules

In a study comprising 32 isolated CAE patients without stenosis, 32 obstructive CAD patients without CAE, and 30 control subjects with normal coronary arteries, Turhan et al. determined that the plasma levels of soluble ICAM-1, VCAM-1, and e-selectin were elevated in the isolated CAE patients compared with the other two groups, suggesting more severe and extensive chronic inflammation in the coronary circulation of CAE patients (Turhan et al., 2005); similar conclusions were drawn by Yilmaz et al. (2006). In subsequent mechanistic studies, adhesion molecules were demonstrated to be involved in the initial stages of inflammation *via* mediation of the adhesion and migration of peripheral blood monocytes to the vascular endothelium and their induction of MMPs to inhibit collagen synthesis, the principal mechanism underlying adhesion molecule involvement in atherosclerosis (Antoniadis et al., 2008).

### Role of C-reactive protein

There are currently contradictory findings in disparate studies regarding the correlation between C-reactive protein (CRP) and CAE. Turhan et al. (2004); Dogan et al. (2016) ascertained that the level of hs-CRP in isolated CAE patients was significantly higher than that observed in obstructive CAD and healthy controls, and speculated that more severe inflammation might be related to the pathogenesis of CAE. Wang et al. found that higher hs-CRP levels were significantly associated with cardiac death and non-fatal myocardial infarction in CAE patients (Wang et al., 2016). In contradistinction, Finkelstein et al. uncovered no difference in CRP levels among CAE, CAD, and normal coronary angiographic patients (Finkelstein et al., 2005). Savino et al. also found that CRP levels were unchanged in patients with CAE or obstructive CAD, or in normal coronary arteries (Savino et al., 2006). Based on the aforementioned research results, it was not possible to generate an exact correlation or a specific mechanism between CRP and CAE, and further research is thus warranted in the future.

### Role of vascular endothelial growth factor (VEGF)

Vascular endothelial growth factor (VEGF) is a signal-inducing molecule that stimulates angiogenesis and plays an important role in inflammatory processes of the vascular system (Antoniadis et al., 2008). Augmented VEGF levels were observed in patients with diffuse CAE (Savino et al., 2006), consistent with neovascularization in the aneurysmal arterial region (Collins et al., 2006). VEGF may be implicated in the pathogenesis and progression of CAE with respect to its induction of MMP synthesis. By contrast, elevated serum VEGF levels may also promote the recovery of damaged blood vessels (Maeno et al., 1998; Savino et al., 2006).

### Role of cytokines

A variety of inflammatory cells, endothelial cells, and fibroblasts produce cytokines that primarily include chemokines, interferons, interleukins (ILs), lymphokines, and tumor necrosis factors (TNFs) that modulate cellular activities through autocrine, paracrine, and endocrine signaling. Small proteins can also act as immunomodulators (Borish and Steinke 2003). It is certain that cytokines play a role in the pathogenesis of CAE, and in recent years authors have proposed that various cytokines secreted from different cell groups promote the pathogenesis of CAE (Li et al., 2009). A report on the immunoinflammatory responses to CAE showed a significant increase in systemic levels of INF-γ, TNF-α, IL-1ß, and IL-8, and lower levels of IL-2 and IL-4 compared with the control group. Although the cytokine environment in CAE is similar to that in CAD, there are marked differences. Levels of IL-6, IL-8, and IL-1ß in CAE patients were significantly elevated relative to patients with CAD, while the levels of IL-2 and IL-4 were significantly attenuated (Boles et al., 2018). This difference suggests that in addition to being similar to the TNF-α-associated Th1-activation pathway in CAD, the low levels of IL-2 in CAE may suggest another, non-atherosclerotic trigger that directly activates the Th1 pathway (El Bakry et al., 2017). With regard to the augmented IL-6 levels observed in CAE patients, it was suggested that activation of smooth muscle cells by IL-6 led to vascular remodeling, and in the absence of M2 macrophages needed to reduce tissue damage, further led to the development of CAE (Ait-Oufella et al., 2011).

## The role of genetics in the pathogenesis of CAE

Genetic variation is reported to be a risk factor in coronary artery disease, and 50 genetic variants that are associated with coronary artery disease have been identified to the present time. However, the genetic mutations associated with coronary artery dilation have received inadequate investigation. Noori et al. identified a familial aggregation of KCNH1 (H member 1 of the potassium voltage-gated channel subfamily) mutations in a rare case of myocardial infarction and coronary dilatation following by diarrhea. KCNH1 is a voltage-gated potassium channel that is primarily expressed in the central nervous system (CNS), and the principal symptom of its mutation is epilepsy; however, the aforementioned patient and his family members did not manifest any associated neurologic signs. This is thus the first-ever report of a genetic mutation associated with CAE (Noori et al., 2019).

Genetic predisposition appears to exert an indirect effect on the development of dilation and is chiefly associated with ACE genotyping or FH. Authors of a retrospective review of 112 patients with CAE or CAD that was only confirmed by coronary angiography detected the ACE ID genotype in both groups and suggested that the DD genotype was a risk factor for CAE (Uyarel et al., 2005). Moreover, novel gene polymorphisms have in recent years been reported to be associated with an elevated incidence of CAE. For example, Yalcin et al. postulated that the c.894G>T polymorphism was a risk factor for CAE (Arif Yalcin et al., 2014). c.894G>T is a nucleotide polymorphism in the eNOS gene associated with eNOS activity, and these authors showed that the presence of the 894T allele increased the risk of CAE 2.8-fold (95% CI = 1.15–6.73; *p* = 0.027). As their T allele frequency was 65% in CAE patients and 38.6% in the control group, they posited that the eNOS gene c.894G> T polymorphism was a risk factor for CAE.

## Conclusion and perspectives

While CAE remains a significant clinical pathology associated with morbidity and mortality (Luo et al., 2017), its exact pathogenesis is not well established. In the pathogenesis of atherosclerosis, the imbalance in lipid metabolism induces inflammatory reactions (Ait-Oufella et al., 2006), and lipid metabolism-related factors also regulate inflammation (Nickel et al., 2009). Abnormal modification and localization of lipoproteins likewise regulate inflammatory reactions (Yu et al., 2015), inflammatory reactions then exacerbate the lipid metabolism imbalance, and inflammation and lipid metabolism disorders subsequently jointly promote the development of atherosclerosis (Andersen et al., 2016). Given that CAE is associated with atherosclerosis in 50% of cases and that disorders in lipid metabolism and abnormal immune and inflammatory reactions in CAE patients are widely reported (Endoh et al., 2004), we speculate that there must be a closer and more prominent relationship between abnormal lipid metabolism and the immune inflammation observed in CAE. Although it is currently not possible to elucidate the exact mechanism(s) subserving CAE due to the multiple contributions by different pathways and the different cell types and molecules involved, discerning the key points within these internal physiologic systems will facilitate the treatment of CAE patients in the future. In addition, novel and powerful lipid-lowering drugs are emerging. However, whether these drugs can inhibit the inflammatory immune responses of patients with CAE and also delay and reverse the process of coronary artery expansion requires further examination.
